# Active Case Finding for Malaria: A 3-Year National Evaluation of Optimal Approaches to Detect Infections and Hotspots Through Reactive Case Detection in the Low-transmission Setting of Eswatini

**DOI:** 10.1093/cid/ciz403

**Published:** 2019-05-16

**Authors:** Michelle S Hsiang, Nyasatu Ntshalintshali, Mi-Suk Kang Dufour, Nomcebo Dlamini, Nomcebo Nhlabathi, Sibonakaliso Vilakati, Calsile Malambe, Zulisile Zulu, Gugu Maphalala, Joseph Novotny, Maxwell Murphy, Alanna Schwartz, Hugh Sturrock, Roly Gosling, Grant Dorsey, Simon Kunene, Bryan Greenhouse

**Affiliations:** 1 Department of Pediatrics, University of Texas Southwestern Medical Center, Dallas; 2 Malaria Elimination Initiative, Global Health Group; 3 Department of Pediatrics, University of California, San Francisco (UCSF); 4 Clinton Health Access Initiative, Eswatini Office, Mbabane; 5 Division of Prevention Science, Department of Medicine, UCSF; 6 Eswatini National Malaria Programme, Manzini; 7 Eswatini National Reference Laboratory, Mbabane; 8 Division of HIV, Infectious Diseases, and Global Medicine, Department of Medicine, UCSF

**Keywords:** malaria elimination, reactive case detection, loop-mediated isothermal amplification, Eswatini, efficiency

## Abstract

**Background:**

Reactive case detection (RACD) is a widely practiced malaria elimination intervention whereby close contacts of index cases receive malaria testing to inform treatment and other interventions. However, the optimal diagnostic and operational approaches for this resource-intensive strategy are not clear.

**Methods:**

We conducted a 3-year prospective national evaluation of RACD in Eswatini, a malaria elimination setting. Loop-mediated isothermal amplification (LAMP) was compared to traditional rapid diagnostic testing (RDT) for the improved detection of infections and for hotspots (RACD events yielding ≥1 additional infection). The potential for index case–, RACD-, and individual-level factors to improve efficiencies was also evaluated.

**Results:**

Among 377 RACD events, 10 890 participants residing within 500 m of index cases were tested. Compared to RDT, LAMP provided a 3-fold and 2.3-fold higher yield to detect infections (1.7% vs 0.6%) and hotspots (29.7% vs 12.7%), respectively. Hotspot detection improved with ≥80% target population coverage and response times within 7 days. Proximity to the index case was associated with a dose-dependent increased infection risk (up to 4-fold). Individual-, index case–, and other RACD-level factors were considered but the simple approach of restricting RACD to a 200-m radius maximized yield and efficiency.

**Conclusions:**

We present the first large-scale national evaluation of optimal RACD approaches from a malaria elimination setting. To inform delivery of antimalarial drugs or other interventions, RACD, when conducted, should utilize more sensitive diagnostics and clear context-specific operational parameters. Future studies of RACD’s impact on transmission may still be needed.

## Teaser

Optimal approaches to find malaria infections and hotspots in reactive case detection are not clear. Here, molecular testing compared to standard rapid diagnostic testing and operational factors (≥80% coverage, response ≤7 days, and 200-m screening radius) maximized efficiency and yield.

To reduce or interrupt malaria transmission, it becomes necessary to find and treat subclinical infections, which can perpetuate transmission [1]. As such, active case finding with treatment in villages of recent passively detected index cases, called reactive case detection (RACD), is widely practiced in low-endemicity settings [2, 3]. The rationale for RACD is that asymptomatic or minimally symptomatic malaria infections cluster in space and time and particularly around households [4, 5]. Several countries partially attribute successful achievement of malaria elimination to deployment of RACD [2, 6]. The identification of hotspots can also inform other community-level interventions such as enhanced case management [7], vector control, or focal mass drug administration [8].

However, RACD can be low yield due to the limited sensitivity of the traditional diagnostics, microscopy or rapid diagnostic testing (RDT); positivity rates are often 0−2% in settings of low endemicity [4, 9–13]. Molecular methods provide improved sensitivity, but polymerase chain reaction (PCR) is not routinely used outside of research because of the technical skill, cost, and long processing times required. Loop-mediated isothermal amplification (LAMP) is an alternative molecular method that offers improved ease of use, shorter processing time, and similar sensitivity to PCR [14]. The available evidence shows PCR or LAMP to increase the detection of actively identified infections by 2-fold compared to RDT or microscopy, but available studies have small sample sizes and limited spatial or temporal scales [4, 10–13, 15, 16]. Also, studies are mainly from Asia and Latin America, where RACD has been practiced for many decades. Due to the historically high endemicity of malaria in most of Africa and recent availability of RDTs, which are generally more convenient than microscopy as a field diagnostic, RACD is a relatively new intervention in Africa. Large-scale evaluations using molecular testing from very low-transmission settings (prevalence <1%, annual incidence <100 per 1000 [17]) are needed.

Operationally, RACD is labor and resource-intensive [10, 18–20]. The required on-call team consumes a significant proportion of a program’s staffing and budget [21]. Determination of the minimum radius size or coverage can inform operational efficiency. However, empirical data of RACD implemented with high coverage and molecular diagnostics beyond the index household are limited [13, 18, 19, 22–24]. A ≤7-day response time is promoted [25], but only 1 study (using RDT) has shown its association with infection detection [24]. Individual-level risk factors for infection among individuals screened (eg, occupation, lack of vector control) are reported [4, 12, 15, 24, 26], but such information does not provide significant opportunities for efficiencies because individuals would still need to be screened to identify such risk factors. Studies have not evaluated index case–level factors, which may inform whether RACD should be triggered. As with active case detection for other communicable diseases such as tuberculosis and leishmaniasis [27, 28], a better understanding of factors associated with improved infection detection would help optimize RACD.

Eswatini (formerly Swaziland) is among several African countries aiming to eliminate malaria [29], and RACD with RDT is implemented in an effort to eliminate residual hotspots of transmission [24, 30]. To understand how to optimize RACD, we aimed (1) to compare LAMP to RDT for improved detection of infections and hotspots and (2) to identify additional epidemiological or operational factors to maximize yield and efficiency.

## METHODS

### Study Design

A prospective population-based surveillance study was conducted through Eswatini’s national passive and reactive malaria case detection program.

### Study Site

Eswatini is a very low-transmission setting with annual malaria incidence of 0.7−1.3 per 1000 population from 2012 to 2015 and parasite prevalence of 0.2% in 2010 [31, 32]. Transmission occurs in the eastern agricultural areas, mainly between October and May. *Plasmodium falciparum* is the primary species and the principal vector is *Anopheles arabiensis*. About half of passively identified cases are considered imported based on travel history, mostly to Mozambique.

### Study Population

Index cases included patients diagnosed by RDT and/or microscopy at any of Eswatini’s 261 public or private health facilities from September 2012 through March 2015 and reported through the national immediate disease notification system. Household members and neighbors residing within 500 m of investigated index cases and residing in the eastern receptive region (receptivity based on altitude and local malaria cases since 2010) were eligible for RACD. Exclusion criteria included RACD performed in the prior 5 weeks (anticipated low likelihood of detecting infections among recently screened and treated individuals) and refusal to participate.

### Data Collection

Data collection was integrated into standard case investigation and RACD, which are implemented by surveillance officers employed and trained by the national malaria program [24, 33]. Within 48 hours of notification, a case investigation was conducted at the index case’s home to collect Global Positioning System coordinates, demographics, clinical history, and epidemiological information including occupation, overnight stays within the prior 1−8 weeks, vector control coverage, and housing quality [34]. For malaria diagnosis quality assurance, a dried blood spot (DBS) was collected for subsequent LAMP testing, if not already collected pretreatment.

Participants residing within 500 m were targeted for RDT testing and DBS collection, and a questionnaire similar to the index case questionnaire was administered in the local language. Household heads were also asked to provide their household population size (and estimates for neighboring households that were vacant) to determine target population size of the RACD event. RDT-positive participants were transported by RACD staff to the nearest health facility for treatment per national policy. If <100% of individuals were reached, return visits were conducted until at least 80% of the estimated population within 500 m was reached. Case investigation or RACD was closed 5 weeks from the date of the index case report. To account for potential ecological confounders of malaria risk, satellite-derived data were collected as previously described [34].

### Laboratory Methods

Rapid diagnostic testing was performed using the First Response *P. falciparum* HRP-2 Detection Test (Premier Medical Corporation Ltd) and microscopy per national guidelines. DNA was Chelex extracted from DBS and 15 µL was used as template for genus-specific LAMP testing and then *P. falciparum*–specific LAMP testing if positive (Loopamp Malaria Pan and Pf Detection Kits, Eiken Chemical Co) [34]. For quality assurance, all LAMP-positive and 10% of LAMP-negative samples underwent genus- and species-specific PCR testing at quarterly intervals. Five microliters of Chelex extraction product was used in nested PCR targeting the cytochrome b gene [32, 35]. As national treatment policies are based on diagnosis by RDT and/or microscopy, LAMP and PCR results were not used to inform treatment.

### Data Management and Analysis

Data were collected on tablet computers, merged into the national Structured Query Language (SQL) database, then cleaned and analyzed in Stata version 13.0 (Stata Corp).

For the detection of infections and hotspots (defined in the study as an RACD event with at least 1 infection found in RACD), the yield and diagnostic accuracy of RDT was compared to LAMP as gold standard. We then measured the relationships between (1) index case– and RACD-level factors and hotspot detection by LAMP and (2) individual-level factors and infections detected by LAMP in RACD. Fisher exact test for 2 × 2 tables or the *t* test for continuous variables was used. Risk ratios were generated using log-binomial regression models and generalized estimating equations with robust standard errors to adjust for clustering at the levels of the household and RACD event. For multivariate models, variables were included a priori or if the *P* value was <.05 in the initial bivariate analysis. Nationality and relationship to the index case were not included due to collinearity with international travel and distance from the index case, respectively. Fever history was not included due to correlation with infection.

**Figure 1.**
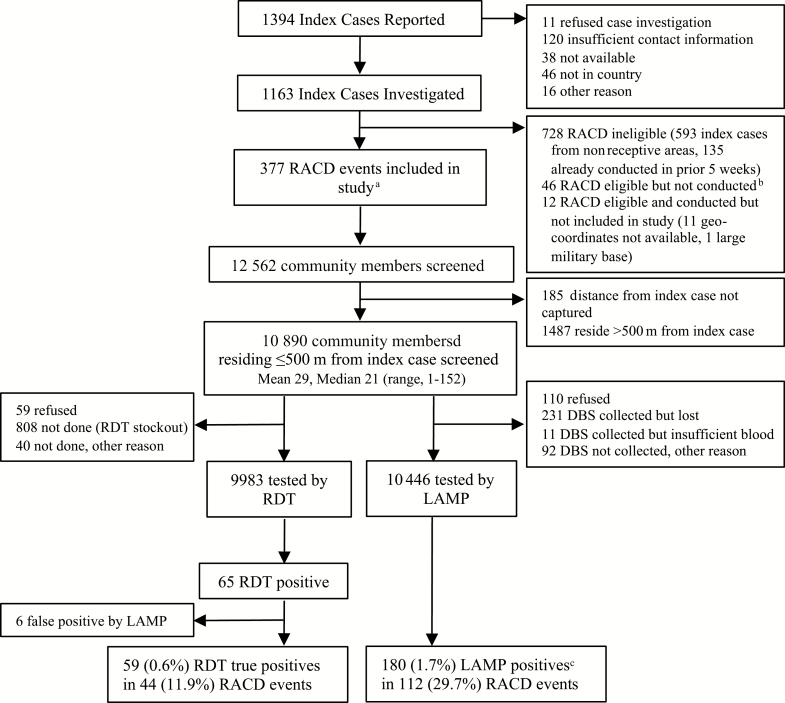
Schematic of reactive case detection (RACD) enrollment and rapid diagnostic test (RDT) vs loop-mediated isothermal amplification (LAMP) results. aOf 350 index cases for whom a DBS was available for confirmatory testing, 262 (74.9%) were confirmed by genus-specific LAMP testing (Pan-LAMP), of which 98.8% (252/255) were confirmed P. falciparum by Pf-LAMP testing. Of 88 LAMP-negative samples, 75% were not collected before treatment or could not be confirmed to have been collected before treatment. bOther reasons RACD not conducted: suspected false positive diagnosis in index case, timed out due to late case reporting or limited staffing, usual residence not in village. cOf these 180 Pan-LAMP positives, 157 (87.2%) were confirmed P. falciparum by Pf-LAMP testing. Abbreviations: DBS, dried blood spot; Pf-LAMP, P. falciparum loop-mediated isothermal amplification.

Finally, compared to a reference of launching RACD in all index cases and screening all individuals residing within 500 m, the potential for more restrictive screening approaches to maximize yield and efficiency to detect hotspots and infections were explored. These included limiting an RACD response to certain index cases, screening by radius or number of households, and staged scenarios whereby further screening was dependent on the presence of at least 1 RDT-positive in the index household or index case–level factors associated with hotspot detection. For each screening approach, the proportions of hotspots detected, RACD events launched, infections detected, and individuals screened were calculated against the reference, and the latter 2 were graphed to visualize yield and efficiencies (Figure 1). Ecological factors were not considered due to variability year-to-year and because the parasite reservoir during high or low seasons can drive transmission [5]. Operational factors were not considered because targets for screening coverage and response time would be predetermined. Individual-level factors were not included because such information would not be available without screening everyone.

### Ethical Considerations

Written informed consent was obtained from participants or a parent or guardian for children <18 years of age. Ethical approval was obtained from the Eswatini Ministry of Health and the University of California, San Francisco.

## RESULTS

### Enrollment

**Figure 2.**
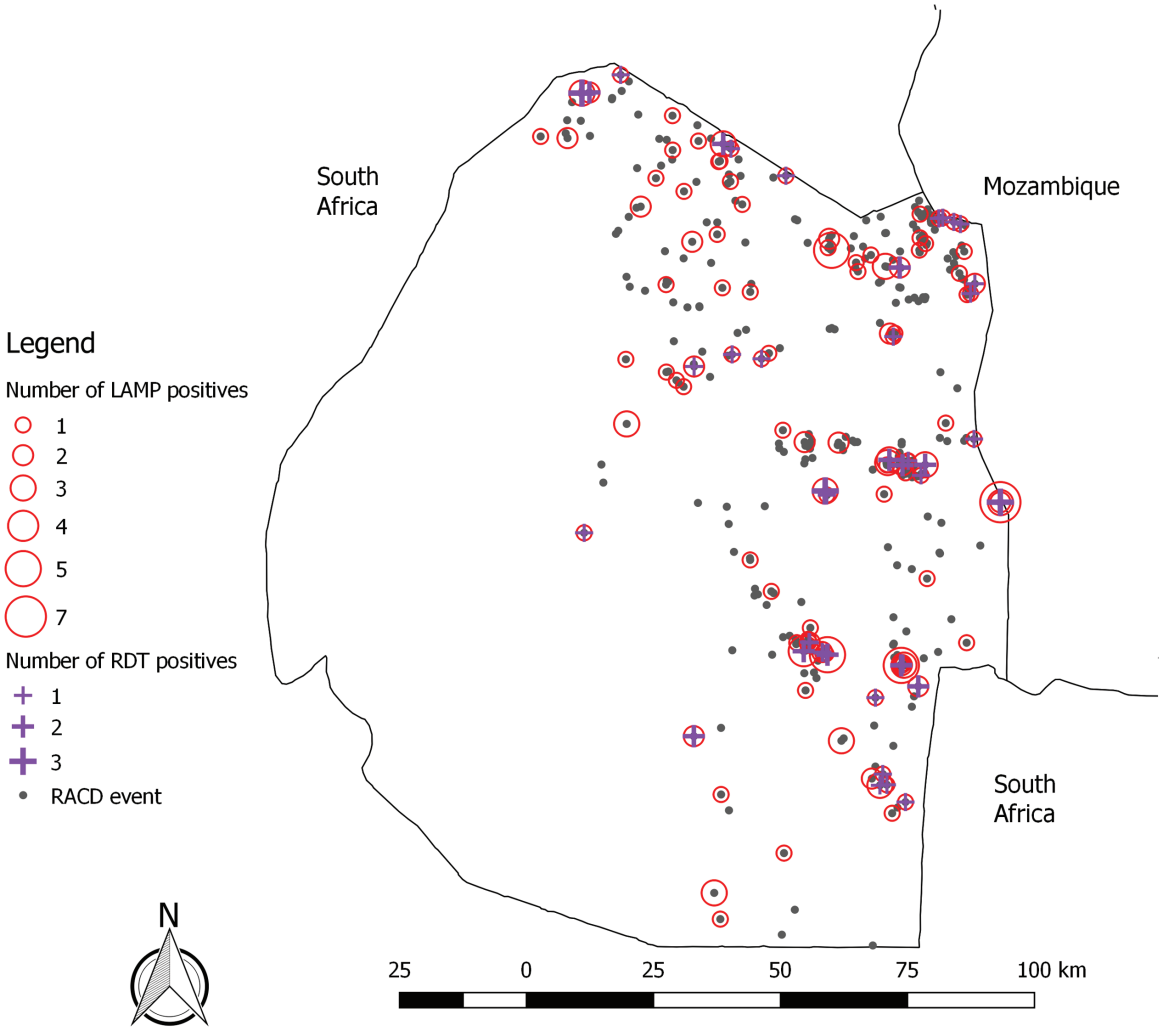
Map of reactive case detection (RACD) events and rapid diagnostic test (RDT)-positive and loop-mediated isothermal amplification (LAMP)-positive infections detected.

Over the study period, 1394 index cases were reported, with most clustered in the northern and eastern regions of the country (Figure 2 and Supplementary Figures 1 and 2). A total of 1163 (83.4%) were investigated, and 377 associated RACD events were conducted. The most common reason case investigation was not performed was insufficient contact information (51.9%) and, for RACD, nonreceptive location (50.4%). Characteristics among index cases and RACD events that were performed were similar to those not performed (Supplementary Tables 1 and 2). In RACD, 10 890 participants were enrolled. Characteristics of index cases, RACD events, and participants screened are shown in Tables 1 and 2 and Supplementary Tables 3 and 4. Index cases were mainly young adult males of Eswatini nationality, with 36.3% reporting international travel and two-thirds not protected by indoor residual spraying (IRS) or a bednet. In RACD, median response time was 5 days and mean coverage of the target population was 62.4%. More than 1 return visit was conducted in 232 (61.5%) of RACD events (median, 2 visits [range, 1–8]). Among participants in RACD, females and children were most highly represented. Median distance from sleeping structure to the index case was 152 m (range, 0–500 m), with 10.6% residing in the index household.

**Table 1.** Multivariable Analysis of Factors Associated With Hotspot Detection (≥1 Versus 0 Loop-mediated Isothermal Amplification-positive Infection in Reactive Case Detection Event), N = 377

Risk Factor	RACD Events, Total (%)	LAMP-positive Hotspots, No. (%)	RR (95% CI)	ARR (95% CI)
Index case–level factors				
Age, y				
<15	111 (29.4)	30 (27.0)	1	1
15–39	185 (49.1)	56 (30.3)	1.12 (.77–1.63)^a^	1.15 (.79–1.69)
≥40	81 (21.5)	26 (32.1)	1.19 (.76–1.85)	1.11 (.70–1.74)
Sex				
Female	104 (27.7)	36 (34.6)	1	1
Male	271 (72.3)	75 (27.7)	0.80 (.58–1.11)	0.67 (.49–.91)^a^
Higher-risk occupation (farming)^b^				
No	329 (88.7)	94 (28.6)	1	1
Yes	42 (11.3)	17 (40.5)	1.42 (.95–2.12)	1.78 (1.24–2.56)^a^
International travel				
None	240 (63.7)	73 (30.4)	1	1
South Africa	16 (4.2)	5 (31.3)	1.03 (.48–2.18)	1.15 (.58–2.25)
Mozambique	116 (30.8)	33 (28.5)	0.94 (.66–1.32)	0.87 (.63–1.20)
Other	5 (1.3)	1 (20.0)	0.66 (.11–3.84)	0.48 (.12–2.01)
Vector control usage				
LLIN and/or IRS	142 (40.2)	75 (35.6)	1	1
Neither	211 (59.8)	33 (23.2)	1.53 (1.08–2.17)^a^	1.71 (1.22–2.39)^a^
Molecular confirmation of RDT or microscopy-based diagnosis^c^				
No (false positive)	22 (5.8)	4 (18.2)	1	1
Yes (true positive)	262 (69.5)	24 (25.8)	1.76 (.71–4.36)	2.02 (.66–6.21)
Not done	93 (24.7)	84 (32.1)	1.42 (.55–3.68)	1.51 (.47–4.90)
Environmental factors				
Season				
Low season	73 (19.4)	29 (39.7)	1	1
High season	304 (80.6)	83 (27.3)	0.69 (.49–.96)	0.78 (.52–1.17)
Land surface temperature, °C, mean (SD)	29.5 (3.1)	28.9 (3.1)	0.96 (.92–1.01)	0.97 (.92–1.03)
Operational factors				
Screening coverage				
<80%	261 (71.3)	66 (25.3)	1	…^d^
≥80%	105 (28.7)	43 (41.0)	1.62 (1.19–2.21)^a^	
Time to RACD, d				
>7	129 (34.3)	24 (18.6)	1	…^d^
≤7	247 (65.7)	88 (35.6)	1.91 (1.29–2.85)^a^	

Abbreviations: ARR, adjusted risk ratio; CI, confidence interval; IRS, indoor residual spraying; LAMP, loop-mediated isothermal amplification; LLIN, long-lasting insecticide-treated net; RACD, reactive case detection; RDT, rapid diagnostic test; RR, risk ratio; SD, standard deviation.

^a^ *P* < .05.

^b^Farming represented 11.3% of all occupations. Lower-risk occupations included manufacturing (2.2%), other manual labor (6.7%), small-market sales or trade (4.6%), office work (5.0%), student (28.6%), unemployed (39.4%), and other (2.2%).

^c^Molecular confirmation of these RDT and/or microscopy-based index cases’ diagnoses was conducted by LAMP. Except for 7 index cases diagnosed by microscopy only (3 true positives and 4 did not have molecular confirmation done), all other diagnoses were by RDT with or without microscopy. Of the 22 false positives, 17 were diagnosed by RDT only and 5 by RDT and microscopy.

^d^Operational factors not included in adjusted model, which aims to identify factors that would inform whether or not RACD should be triggered.

### Laboratory Results

**Figure 3.**
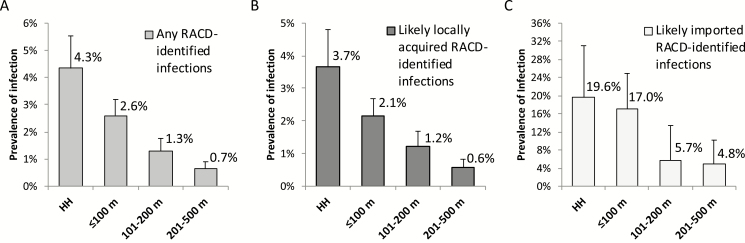
Prevalence of loop-mediated isothermal amplification-positive infection by distance from index case among all participants screened in reactive case detection (n = 10 890) (A), limited to Eswatini nationals without recent travel to Mozambique (ie, at risk of locally acquired infection, n = 10 215) (B), and limited to Mozambicans or participants with recent travel to Mozambique (ie, at risk of imported malaria, n = 231) (C). Abbreviations: HH, household; RACD, reactive case detection.

Overall and by year and season, LAMP identified more infections and hotspots, including all that were identified by RDT (Figures 2 and 3 and Supplementary Figures 1 and 2). Compared to RDT, LAMP provided a 3-fold higher yield to detect infections (1.7% vs 0.6%) and a 2.3-fold higher yield to detect hotspots (29.7% vs 12.7%). Compared to the number of passively detected cases in receptive areas (n = 377), the 180 infections identified by RACD using LAMP increased the detection of infections by 47.7% (compared to 15.6% with RACD using RDT, which identified 59 additional infections). Of note, RDT did not identify any of the LAMP-detected infections or hotspots in the low season of year 2 (Supplementary Figures 1 and 2).

### Diagnostic Accuracy of RDT Using LAMP as Gold Standard in RACD

To detect hotspots, the sensitivity and negative predictive value of RDT using LAMP as gold standard were 40.5% (95% confidence interval [CI], 35.5%–45.5%) and 79.6% (95% CI, 75.5%–83.7%), respectively; among participants screened in RACD, they were 33.4% (95% CI, 32.8%–34.7%) and 98.8% (95% CI, 98.6%–99.0%), respectively. Specificity and positive predictive value were both >90%. Using PCR as gold standard for quality assurance in a subsample, sensitivity of LAMP was 72.2% (95% CI, 62.6%−80.2%) (3 of 406 LAMP-negatives were PCR positive) and specificity was 98.0% (95% CI, 97.5%−98.4%).

### Factors Associated With Hotspot Detection

In the adjusted analysis, several index case–level factors (female sex, farming, lack of vector control usage) were associated with hotspot detection (Table 1). Compared to false-positive index case diagnoses, the adjusted risk ratio (ARR) for true-positive index case diagnoses was high, but the strength of the association was weak (2.02 [95% CI, 0.66−6.21]). Operational factors such as ≥80% coverage of the target population and RACD implementation within ≤7 days of index case reporting had RRs of 1.62 and 1.91, respectively.

### Factors Associated With LAMP-positive Infection Among Individuals Screened in RACD

**Figure 4.**
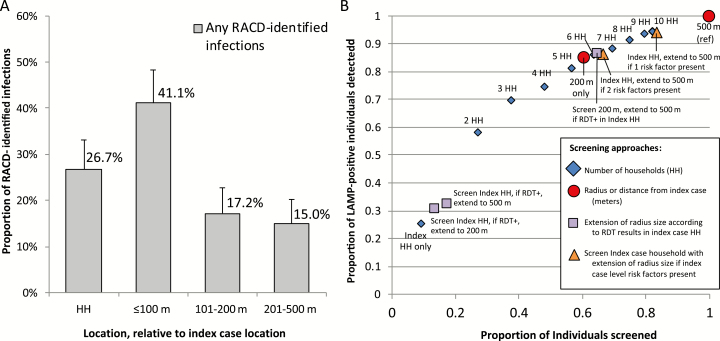
A, Proportion of LAMP-positive infections by distance from index case (n = 180). B, Yield and efficiencies of different reactive case detection scenarios compared to a reference of screening all participants residing within 500 m of the index case. Abbreviations: HH, household; LAMP, loop-mediated isothermal amplification; RACD, reactive case detection; RDT, rapid diagnostic test.

Distance from index case was the strongest individual-level predictor of infection (Table 2). Compared to residence at 201–500 m, residence at 101–200 m, within 100 m but not in the same household, and within the same household had an ARR of 2.00, 3.14, and 4.13, respectively. A higher prevalence of LAMP-detectable infection among participants residing closer to index cases was present whether or not infection was likely acquired outside or within Eswatini (Figure 4). Certain occupations (farming, manual labor, or small market sales), medium- or low-quality housing, and lack of vector control were also associated. Travel to Mozambique was strongly associated with infection (ARR, 9.99), though only 1.7% of participants reported international travel.

**Table 2.** Multivariable Analysis of Factors Associated With Infection Detection Among Individuals Screened (Loop-mediated Isothermal Amplification [LAMP] Positive Versus LAMP Negative), N = 10 446

Individual-level Factors	Screened in RACD, Total (%)	LAMP-positive Infections, No. (%)	RR (95% CI)	ARR (95% CI)
Age, y				
<15	4623 (44.3)	69 (1.5)	1	1
15–39	3754 (35.9)	79 (2.1)	1.41 (1.02–1.94)^a^	1.27 (.90–1.79)
≥40	2069 (19.8)	32 (1.6)	1.04 (.68–1.57)	0.95 (.60–1.50)
Sex				
Female	6047 (57.9)	95 (1.6)	1	1
Male	4399 (42.1)	85 (1.9)	1.23 (.92–1.64)	1.24 (.92–1.68)
Higher-risk occupation (farming, manual labor, small market sales)^b^				
No	9294 (89.4)	147 (1.6)	1	1
Yes	1102 (10.6)	33 (3.0)	1.89 (1.30–2.75)^a^	1.60 (1.05–2.44)^a^
International travel				
None	10122 (96.9)	151 (1.5)	1	1
South Africa	146 (1.4)	3 (2.1)	1.38 (.45–1.45)	1.34 (.42–4.28)
Mozambique	173 (1.7)	25 (14.5)	9.69 (6.52–14.39)^a^	9.99 (6.55–15.24)^a^
Other	5 (0.1)	1 (20.0)	13.41 (2.31–77.94)^a^	20.65 (2.55–167.03)^a^
Housing quality				
High	6998 (68.0)	95 (1.4)	1	1
Medium	2704 (26.3)	68 (2.5)	1.87 (1.38–2.55)^a^	1.74 (1.29–2.35)^a^
Low	594 (5.8)	16 (2.7)	2.01 (1.19–3.38)^a^	1.95 (1.16–3.29)^a^
Vector control usage				
LLIN and/or IRS	5781 (58.3)	131 (2.3)	1	1
Neither	4139 (41.7)	43 (1.0)	2.16 (1.56–2.99)^a^	1.80 (1.29–2.50)^a^
Distance from index case, m				
201–500	4121 (39.5)	27 (0.7)	1	1
101–200	2377 (22.8)	31 (1.3)	1.99 (1.19–3.33)^a^	2.00 (1.20–3.35)^a^
≤100	2840 (27.2)	74 (2.6)	3.98 (2.57–6.16)^a^	3.14 (2.01–4.91)^a^
Same household	1108 (10.6)	48 (4.3)	6.61 (4.14–10.55)^a^	4.13 (2.57–6.62)^a^
Relationship to index case				
Neighbor	8773 (84.0)	97 (1.1)	1	…^c^
Family	1667 (16.0)	82 (4.9)	4.45 (3.33–5.94)^a^	

Abbreviations: ARR, adjusted risk ratio; CI, confidence interval; IRS, indoor residual spraying; LAMP, loop-mediated isothermal amplification; LLIN, long-lasting insecticide-treated net; RACD, reactive case detection; RR, risk ratio.

^a^ *P* < .05.

^b^Farming, manual labor, and small market sales represented 6.7%, 2.2%, and 1.6% of all occupations. Lower-risk occupations included manufacturing (0.6%), office work (1.0%), student (28.3%), unemployed (57.6%), and other (2.0%).

^c^Not included in multivariate model due to correlation with distance from index case.

### Optimizing RACD

Beyond coverage and response time, we explored the potential for other screening strategies to maximize yield and efficiency to detect hotspots and infections, compared to a reference of launching RACD in all index cases and screening all individuals within 500 m. Restricting RACD to index cases with at least 1 epidemiological risk factor (female, farming, or lack of vector control), while maintaining a screening radius of 500 m within those RACD events, prevented 24.1% of RACD events from being launched and 25.2% of individuals from screening, and identified 82.1% and 80% of hotspots and infections, respectively. If RACD were launched for all index cases, restricting screening to the index household residents prevented 90.1% of individuals from screening and identified 26.7% of infections (Figure 1) and 37.5% of hotspots; extending to 200 m or the 6 closest households saved almost 40% of individuals from screening and detected >85% of infections and 92% of hotspots. Screening beyond 200 m, based on RDT positivity in the individual–, household-, or index case–level factors provided incremental increases in yield with increased operational complexity.

## DISCUSSION

RACD is a widely practiced and resource intensive malaria active case finding intervention with limited evidence to guide best practice in elimination settings [10, 18, 21, 36]. In this large-scale national study covering 3 transmission seasons, RDTs had limited sensitivity for the detection of infections and hotspots in RACD. Hotspot detection was associated with high coverage of the target population and timely response. There was a dose-dependent relationship between distance from index cases, and limiting RACD to 200 m maximized yield and efficiency. Other epidemiological factors were associated with infection, but such information did not provide opportunities for improved yield or efficiency in RACD. These findings highlight the importance of implementing quality and tailored approaches for surveillance and to efficiently target populations at highest risk of malaria. Findings also point to the need for more sensitive point-of-care diagnostics [37] or improved systems to enable molecular results to inform individual-level treatment or hotspot-level intervention response.

RACD is increasingly being included in malaria elimination strategies of African countries [38], but there are concerns about the limited sensitivity of current diagnostics, and scarce data on impact and operations [39]. From very low-transmission African settings, RACD evaluations have mainly used RDT [24, 26, 40], with only 2 smaller-scale studies utilizing molecular testing [4, 16]. In our LAMP-based evaluation of 10 890 participants screened in RACD over 3 years, we confirm the low sensitivity of RDT (33.7%). We also found low sensitivity of RDT to detect hotspots (40.5%), which has previously not been reported.

Beyond the diagnostic test, operational approaches can be exploited to maximize yield to detect infections. Consistent with other observational studies, we found a higher risk of infection in index case households [4, 10, 11]. The added value of screening beyond the index household has not been clear. A 1-km radius was previously recommended based on the reasonable distance that mosquitoes fly [41]. Our finding that risk declines beyond 200 m supports recent policies in southern Africa that limit RACD to ≤200 m due to feasibility [24, 42]. Screening limited to the closest 6 households provided a similar yield, but this approach may not be generalizable given differences in housing or population density. Our finding that screening within 7 days was associated with hotspot detection is consistent with a prior study based on RDT [24] and supports policies promoted by China [25]. As 1 month is generally necessary for transmission from one person to another, it also suggests that “index cases” may represent a peak in the focal spread of infection, and not the source of the outbreak. Finally, and consistent with recommendations, ≥80% target population coverage was associated with hotspot detection [41].

In RACD, individuals with certain occupations, international travel, low-quality housing, lack of vector control, fever, and history of malaria were more likely to be infected. While these individuals could be prioritized for malaria testing, individual-level screening for presence of risk factors would still be required, thus compromising operational efficiency. Furthermore, the strongest risk factors such as international travel (Table 2) were only present in a small proportion of RACD-identified cases. As such, index case–level factors were assessed to inform whether RACD should be triggered at all. Factors likely associated with local transmission (lack of travel and vector control in the index case) were associated with hotspot detection. Female sex of the index case was also associated, and may reflect local transmission if females in Eswatini are less likely to have travel and occupational risk factors for infection [43]. However, a simple approach of using a 200-m screening radius around all index cases without consideration of index case–level factors maximized yield and efficiency of (Figure 1). In the future, or in other settings, index case–level factors may be more relevant. For example, compared to the 5.8% of RACD events that were triggered by a false-positive index case diagnoses by RDT and/or microscopy, LAMP-confirmed true-positive index case diagnoses were twice as likely to result in hotspot identification (Table 1). Although the strength of the association was weak, false-positive index case diagnoses may become more common as transmission declines [44, 45], and molecular confirmation before launching RACD could be considered.

A potential weakness of the study is that 7.5% of participants did not receive RDT testing due to a stockout. However, there was unlikely introduction of bias as analyses were largely based on LAMP, which was conducted in 96.1% of eligible participants. Second, the sensitivity of LAMP was 72.2% relative to nested PCR; however, the small number of LAMP-negative PCR-positive samples (n = 3) could be explained by stochastic variation. Finally, the main analysis included infections irrespective of travel history, which may suggest that findings do not reflect dynamics of local transmission. However, imported cases in receptive areas can contribute to local transmission. Furthermore, the effect of travel history was considered in stratified and adjusted analyses.

Our study had several strengths. As the first large-scale national evaluation of RACD from a very low-transmission African setting, our study fills a critical gap in the existing literature. Most available studies are based on RDT or microscopy, and we utilized molecular methods to include subpatent infections. Even in settings where use of molecular methods to inform treatment of individual is impractical, it can be used as a surveillance tool to identify hotspots. More sensitive point-of-care diagnostics are also becoming available [37]. Our analysis was also more comprehensive and thus operationally relevant than other studies, as we assessed index case– and RACD-level risk factors for hotspot detection, in addition to individual-level risk factors. Also, in other studies, RACD coverage beyond the index household has not been uniform or reported, thus limiting assessment of risk beyond the index household [13]. The extent to which more distant infections are related to the index case is currently being evaluated with genotyping.

When implemented, RACD should be conducted with more sensitive diagnostics and clear context-specific operational parameters. By enabling detection of infections and hotspots that would otherwise not be detected through passive surveillance, RACD can support efficient delivery of drugs to individuals, and community-level interventions (eg, IRS or mass drug administration) to hotspots. Optimizing the yield and efficiency of RACD may enable better targeting of limited resources for maximal impact [46]. Although temporal trends did not show declines in malaria cases and hotspots (Supplementary Figures 1 and 2), the study was not designed to assess such impact. Treatment of additional infections and targeting of interventions based on acquired surveillance information about hotspots would be expected to reduce or prevent increases in transmission [1], but to address this question, trials comparing RACD to no RACD or alternative approaches such as presumptive treatment may be needed.

## Supplementary Data

Supplementary materials are available at *Clinical Infectious Diseases* online. Consisting of data provided by the authors to benefit the reader, the posted materials are not copyedited and are the sole responsibility of the authors, so questions or comments should be addressed to the corresponding author.

ciz403_Suppl_Supplementary_Material

## Notes

***Author contributions.*** S. K. and M. S. H. conceived the study; M. S. H., B. G., N. Nt., S. K., J. N., and G. D. designed the study; N. D., S. K., and Z. Z. supervised the data collection with support from N. Nt., C. M., J. N., S. V., and M. S. H.; N. Nh., M. M., and A. S. performed laboratory testing with guidance from G. M., M. S. H., and B. G.; S. V., M. S. H., and M. K. D. cleaned the data and performed the data analysis; H. S. supported the spatial analyses; N. D., S. K., N. Nt., R. G., G. D., and B. G. provided additional input on the data analysis; and M. S. H. wrote the manuscript. All authors read and approved the final manuscript.

***Acknowledgments.*** The authors are grateful for participation of the community and data collection support from the surveillance team of the national program; Glenys Sikhondze, Sindisiwe Dlamini, Iveth J. González, and David Bell for their support on the loop-mediated isothermal amplification assays; Alemayehu Midekisa for gathering ecological data; and Brooke Whittemore for assistance with formatting and the figures. The authors also thank Robert Haley, Jay Tureen, and Richard Feachem for their guidance, and Eliza Walwyn-Jones and George Shirreff for their review of the paper.

***Financial support.*** This work was primarily supported by the National Institutes of Health/National Institute of Allergy and Infectious Diseases (grant number K23AI101012); the Burroughs Wellcome Fund/American Society of Tropical Medicine and Hygiene (grant number A120079); and the Eswatini Ministry of Health. Additional support was provided by the Bill & Melinda Gates Foundation to the University of California San Francisco (UCSF) (grant number A121292) the Clinton Health Access Initiative (CHAI) as a subgrantee of UCSF, and Eswatini National Malaria Program as a subgrantee of CHAI. Additional funding was provided by the Horchow Family Fund Scholarship at the University of Texas, Southwestern. and the Horchow Family Fund Scholarship Award (grant number 5300375400).

***Potential conflicts of interest.*** The authors: No reported conflicts of interest. All authors have submitted the ICMJE Form for Disclosure of Potential Conflicts of Interest. Conflicts that the editors consider relevant to the content of the manuscript have been disclosed.
