# Supplementary material for: Active Case Finding for Malaria: A 3-Year National Evaluation of Optimal Approaches to Detect Infections and Hotspots Through Reactive Case Detection in the Low-transmission Setting of Eswatini
Source: Clin Infect Dis. 2019 May 16;70(7):1316–25. doi: 10.1093/cid/ciz403 (PMC7318780; doi:10.1093/cid/ciz403)
Supplement: ciz403_Suppl_Supplementary_Material [file ciz403_suppl_supplementary_material.docx]

Supplemental table 1. Characteristics of index cases that received and did not receive a case investigation

|  | | Case investigation conducted  (N=1,163)  No. (%) | Case investigation not conducted  (N=231)  No. (%) |
| --- | --- | --- | --- |
| Index case-level factors | |  |  |
| Age | <15 years  15-39 years  ≥40 years | 342 (29.4)  590 (50.7)  231 (19.9) | 76 (32.9)  104 (45.0)  51 (22.1) |
| Gender | Female  Male  Not available | 280 (24.1)  880 (75.7)  3 (0.3%) | 72 (31.2)  158 (68.4)  1 (0.4) |
| Environmental factors |  |  |  |
| Season | Low season  High season | 187 (16.1%)  976 (83.9%) | 26 (11.3)  205 (88.7) |

Note: Among index cases that did not receive a case investigation, only data on age, gender and season were available from case reports. Data on other variables (e.g. occupation, travel, etc) were not available due lack of case investigation.

Supplemental table 2. Characteristics of RACD events that were conducted and those that were eligible but not conducted

|  | | RACD events conducted  (N=337)  No. (%) | RACD eligible but not conducted  (N=46)  No. (%) |
| --- | --- | --- | --- |
| Index case-level factors | |  |  |
| Age | <15 years  15-39 years  ≥40 years | 111 (29.4)  185 (49.1)  81 (21.5) | 10 (21.7)  24 (52.2)  12 (26.1) |
| Gender | Female  Male | 104 (27.7)  271 (72.3) | 15 (32.6)  31 (67.4) |
| Higher risk occupation (farming)† | No  Yes | 329 (88.7)  42 (11.3) | 39 (86.7)  6 (13.3) |
| International travel | None  South Africa  Mozambique  Other | 240 (63.7)  16 (4.2)  116 (30.8)  5 (1.3) | 26 (56.5)  2 (4.4)  16 (34.8)  2 (4.4) |
| Vector control usage | LLIN and/or IRS  Neither | 142 (40.2)  211 (59.8) | 12 (30.8)  27 (69.2) |
| Molecular confirmation of RDT or microscopy-based diagnosis‡ | No (false positive)  Yes (true positive)  Not done | 22 (5.8)  262 (69.5)  93 (24.7) | 1 (2.2)  20 (43.5)  25 (54.4) |
| Environmental factors |  |  |  |
| Season | Low season  High season | 73 (19.4)  304 (80.6) | 6 (13.0)  40 (87.0) |
| Land surface temperature, mean (SD) | ºC | 29.5 (3.1) | 29.1 (2.5) |

Supplemental table 3. Characteristics of RACD events with at least one or more LAMP-positive versus none

| Index case or RACD-level variable | | | No LAMP+ | ≥1 LAMP+ | p-value |
| --- | --- | --- | --- | --- | --- |
| Index case-level epidemiological factors | Age, No. (%) | <15 years  15-39 years  ≥40 years | 81 (73.0)  129 (69.7)  55 (67.9) | 30 (27.0)  56 (30.3)  26 (32.1) | 0.729 |
|  | Gender, No. (%) | Female  Male | 68 (65.4)  196 (72.3) | 36 (34.6)  75 (27.7) | 0.187 |
|  | Nationality, No. (%) | Eswatini  South African  Mozambican  Other | 237 (71.6)  2 (11.8)  20 (100)  5 (71.4) | 94 (28.4)  15 (88.2)  0 (0)  2 (28.6) | 0.259 |
|  | Higher risk occupation (farming), No. (%)† | No  Yes | 235 (71.4)  25 (59.5) | 94 (28.6)  17 (40.5) | 0.113 |
|  | International travel, No. (%) | None  South Africa  Mozambique  Other | 167 (69.6)  11 (68.8)  83 (71.6)  4 (80.0) | 73 (30.4)  5 (31.3)  33 (28.5)  1 (20.0) | 0.942 |
|  | Domestic travel, No. (%) | No  Yes | 233 (70.4)  32 (69.6) | 98 (29.6)  14 (30.4) | 0.908 |
|  | Indoor Residual Spray (IRS) coverage, No. (%) | No  Yes | 161 (65.5)  80 (77.7) | 85 (34.6)  23 (22.3) | 0.024* |
|  | Long-lasting insecticidal net (LLIN) use, No. (%) | No  Yes | 214 (68.2)  50 (82.0) | 100 (31.9)  11 (18.0) | 0.031* |
|  | Vector control usage, No. (%) | LLIN and/or IRS  Neither | 136 (64.5)  109 (76.8) | 75 (35.6)  33 (23.2) | 0.014* |
|  | Housing quality, No. (%) | High  Medium  Low | 178 (72.4)  59 (65.6)  21 (75.0) | 68 (27.6)  31 (34.4)  7 (25.0) | 0.422 |
|  | Household size, No. (%) | mean (SD) | 6.9 (4.3) | 7.8 (5.3) | 0.076 |
| Index case-level clinical factors | Initial diagnostic method, No. (%) | Microscopy only  RDT only  RDT and microscopy | 7 (100)  164 (70.1)  93 (69.9) | 0 (0)  70 (29.9)  40 (30.1) | 0.226 |
|  | Molecular confirmation of diagnosis, No. (%) | No (false positive)  Not done  Yes (true positive) | 18 (81.8)  69 (74.2)  178 (67.9) | 4 (18.2)  24 (25.8)  84 (32.1) | 0.250 |
|  | Disease severity, No. (%) | Uncomplicated  Severe  Unknown | 208 (70.8)  53 (68.8)  4 (66.7) | 86 (29.3)  24 (31.2)  2 (33.3) | 0.903 |
|  | Treatment, No. (%)‡ | AL only  IV/IM quinine  Other | 194 (69.0)  51 (71.8)  20 (80.0) | 87 (31.0)  20 (28.2)  5 (20.0) | 0.492 |
|  | Primaquine, No. (%) | No primaquine  Primaquine | 255 (70.6)  8 (61.5) | 106 (29.4)  5 (38.5) | 0.480 |
|  | Time to seeking care, No. (%) | Mean days (SD) | 3.9 (3.9) | 4.1 (3.2) | 0.693 |
|  | Malaria diagnosis in past year, No. (%) | No  Yes | 240 (70.0)  24 (75.0) | 103 (30.0)  8 (25.0) | 0.551 |
| RACD-level ecological factors | Malaria transmission season, No. (%) | Low season (Jun−Sep)  High season (Oct−May) | 44 (60.3)  221 (72.7) | 29 (39.7)  83 (27.3) | 0.037* |
|  | Transmission year, No. (%) | Sept 2012−Jun 2013  Jul 2013−Jun 2014  Jul 2014−Mar 2015 | 41 (70.7)  127 (75.6)  97 (64.2) | 17 (29.3)  41 (24.4)  54 (35.8) | 0.086 |
|  | Region, No. (%) | Hhohho  Lubombo  Manzini  Shiselweni | 49 (68.1)  200 (72.2)  11 (57.9)  5 (55.6) | 23 (31.9)  77 (27.8)  8 (42.1)  4 (44.4) | 0.393 |
|  | Water body distance, meters, No. (%) | >1000  101−1000  ≤100 | 86 (72.3)  78 (71.6)  97 (68.8) | 33 (27.7)  31 (28.4)  44 (31.2) | 0.808 |
|  | Remote sensing data covariates, mean (SD)§ | Land surface temperature (°C)  Normalized difference water index (-1 to 1)  Elevation (10 m) | 29.4 (3.1)  -0.07 (0.07)  36.9 (15.7) | 28.9 (3.1)  -0.07 (0.07)  35.0 (15.6) | 0.154  0.378  0.306 |
| RACD-level operational factors | Screening coverage, No. (%) | <80%  ≥80% | 195 (74.7)  62 (59.1) | 66 (25.3)  43 (41.0) | 0.003* |
|  | Time to RACD, No. (%) | >7 days  ≤7 days | 105 (81.4)  159 (64.4) | 24 (18.6)  88 (35.6) | 0.001* |

LAMP loop-mediated isothermal amplification; RR risk ratio; ARR adjusted risk ratio; SD standard deviation; RACD reactive case detection

* p<0.05

† Initial analyses showed farming to confer a higher risk of hot spot detection. Farming represented 11.3% of all occupations. Other occupations included: manufacturing (2.2%), other manual labor (6.7%), and small-market sales or trade (4.6%), office work (5.0%), student (28.6%), unemployed (39.4%), and other (2.2%). A binary variable (high relative to low risk) was utilized for subsequent analyses.

‡IM/IV quinine +/- AL or oral quinine; Other: oral quinine 13, AL+ oral quinine 9, none 1

§Of the 3 remote sensing ecological data, none were associated with infection detection in RACD. Land surface temperature was included in the multivariate model (Table 1) due to previous findings in this setting [[32](#_ENREF_32)].

Supplemental table 4. Characteristics of LAMP-positive versus LAMP-negative individuals screened in RACD

| Individual-level variable | | | LAMP neg | LAMP+ | p-value |
| --- | --- | --- | --- | --- | --- |
| Epidemiological factors | Age, No. (%) | <15 years  15-39 years  ≥40 years | 4554 (98.5)  3675 (97.9)  2037 (98.5) | 69 (1.5)  79 (2.1)  32 (1.6) | 0.080 |
|  | Gender, No. (%) | Female  Male | 5952 (98.4)  4314 (98.1) | 95 (1.6)  85 (1.9) | 0.161 |
|  | Nationality, No. (%) | Eswatini  South African  Mozambican  Other | 10153 (98.4)  6 (100)  72 (90.0)  35 (92.1) | 169 (1.6)  0 (0)  8 (10)  3 (7.9) | <0.001* |
|  | Higher risk occupation (farming, manual labor, small market sales), No. (%)† | No  Yes | 9147 (98.4)  1069 (97.0) | 147 (1.6)  33 (3.0) | 0.001* |
|  | International travel, No. (%) | None  South Africa  Mozambique  Other | 9971 (98.5)  143 (98.0)  148 (85.6)  4 (80.0) | 151 (1.5)  3 (2.1)  25 (14.5)  1 (20.0) | <0.001* |
|  | Domestic travel, No. (%) | No  Yes | 9768 (98.3)  498 (98.6) | 173 (1.7)  7 (1.4) | 0.551 |
|  | Indoor Residual Spray (IRS) coverage, No. (%) | No  Yes | 6121 (97.8)  3521 (99.0) | 139 (2.2)  35 (1.0) | <0.001* |
|  | Long-lasting insecticidal net (LLIN) use, No. (%) | No  Yes | 8890 (98.1)  1376 (99.3) | 170 (1.9)  10 (0.7) | 0.002* |
|  | Vector control usage, No. (%) | LLIN and/or IRS  Neither | 5650 (97.7)  4096 (99.0) | 131 (2.3)  43 (1.0) | <0.001* |
|  | Housing quality, No. (%) | High  Medium  Low | 6903 (98.6)  2636 (97.5)  578 (97.3) | 95 (1.4)  68 (2.5)  16 (2.7) | <0.001* |
|  | Relationship to index case, No. (%) | Neighbor  Family | 8676 (98.9)  1585 (95.1) | 97 (1.1)  82 (4.9) | <0.001* |
|  | Distance from index case (m), No. (%) | 201-500 m  101-200 m  ≤100 m  Same household | 4094 (99.3)  2346 (98.7)  2766 (97.4)  1060 (95.7) | 27 (0.7)  31 (1.3)  74 (2.6)  48 (4.3) | <0.001* |
| Clinical factors | Fever in last 2 weeks, No. (%) | No  Yes | 10171 (98.3)  95 (94.1) | 174 (1.7)  6 (5.9) | 0.001* |
|  | Malaria diagnosis in the past year, No. (%) | No  Yes | 10218 (98.3)  48 (90.3) | 175 (1.7)  5 (9.4) | <0.001* |

LAMP loop-mediated isothermal amplification; RACD reactive case detection

* p<0.05

† Initial analyses showed certain occupations to confer a higher risk of hot spot detection: farming, manual labor, and small market sales which represented 6.7%, 2.2%, and 1.6% of all occupations. Lower risk occupations included: manufacturing (0.6%), office work (1.0%), student (28.3%), unemployed (57.6%), and other (2.0%). A binary variable (high relative to low risk) was utilized for subsequent analyses

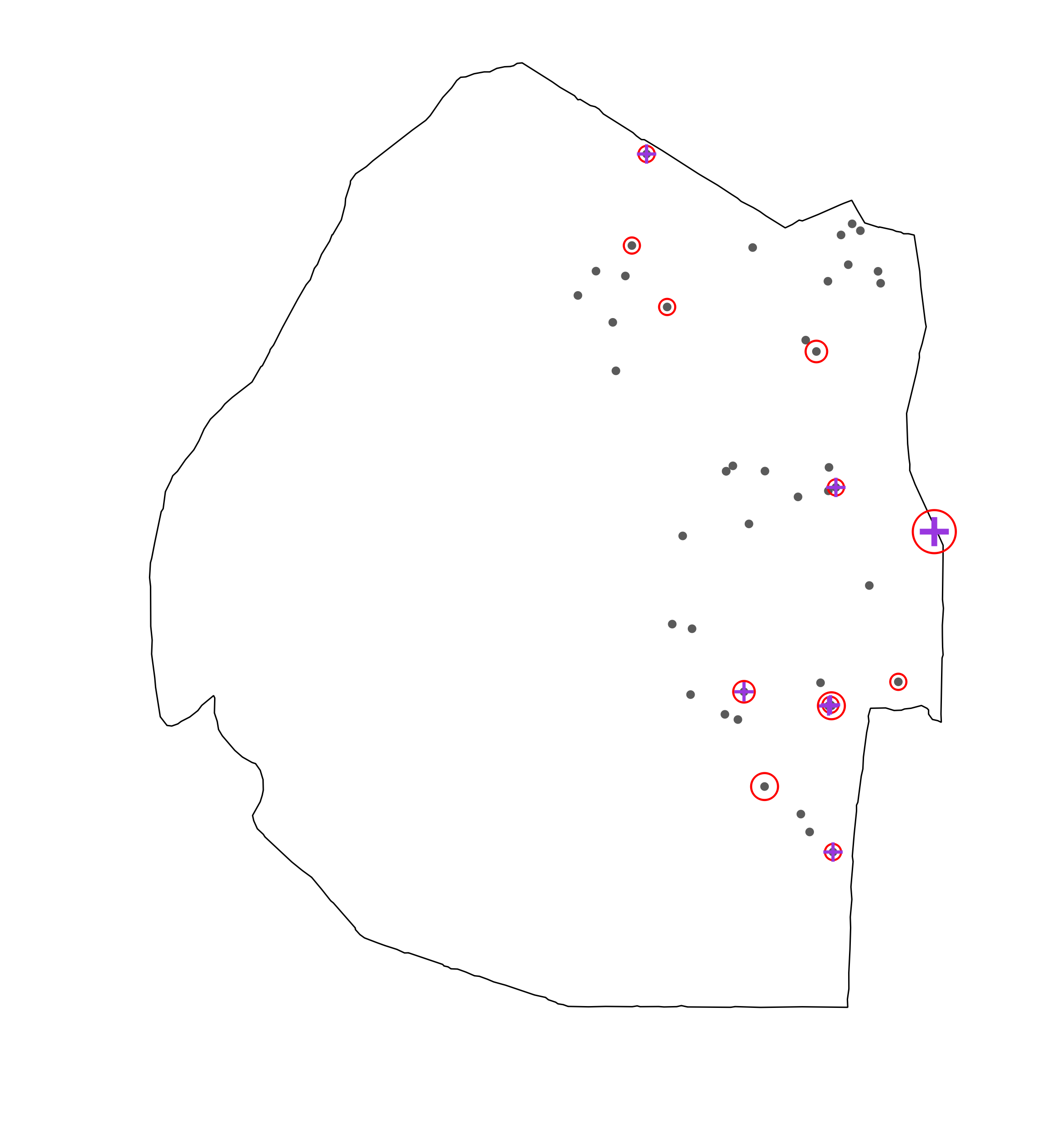

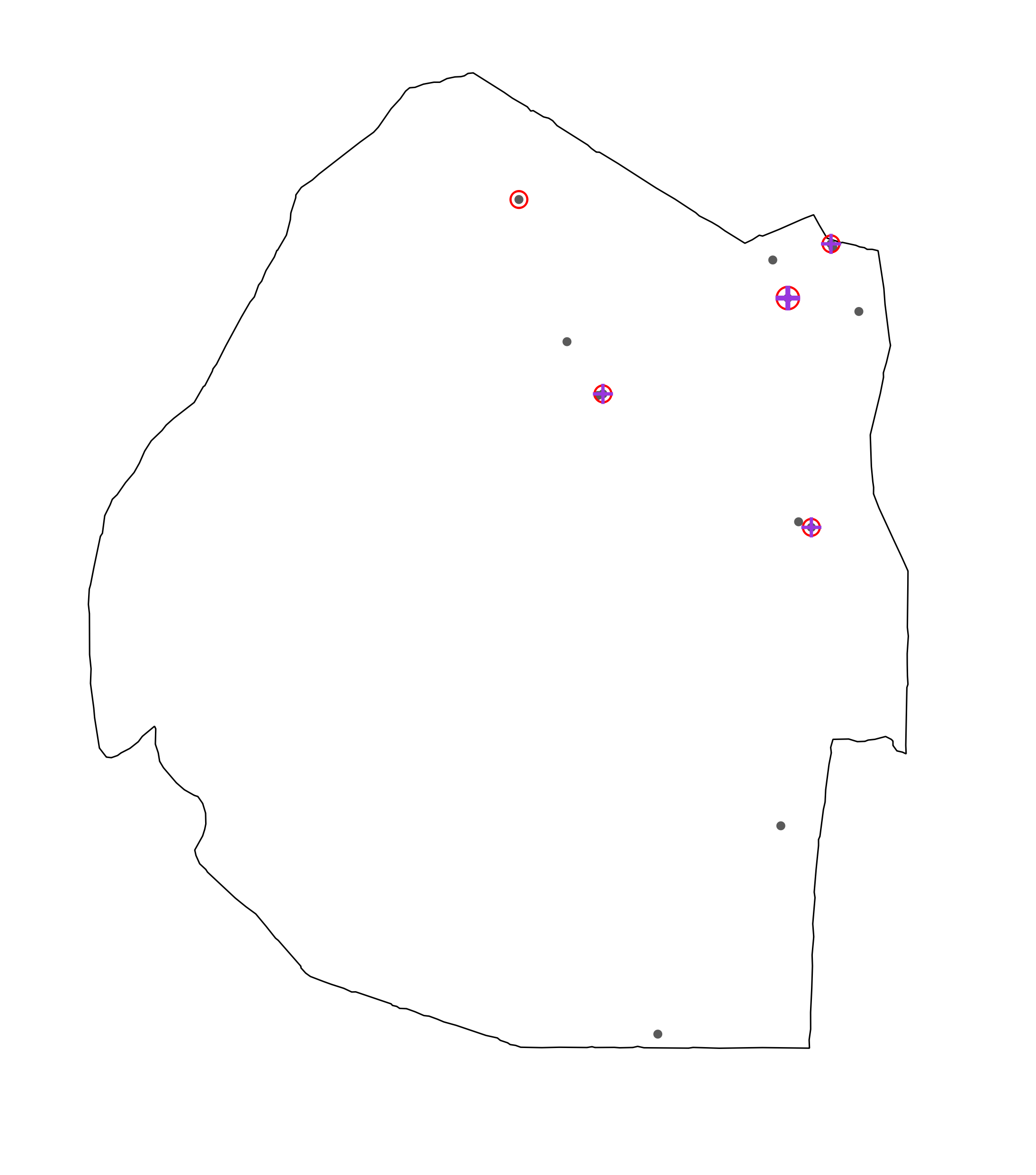

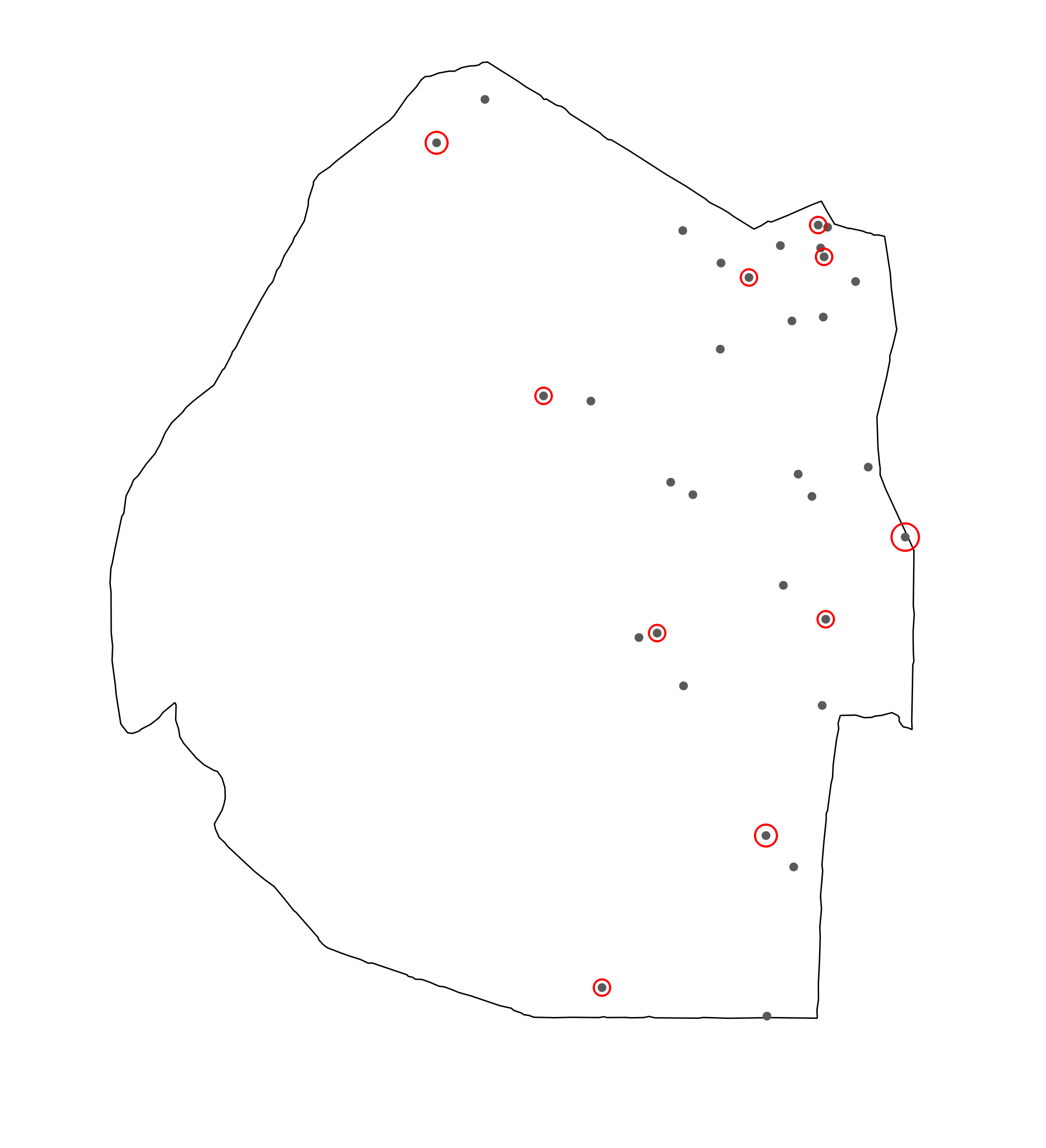

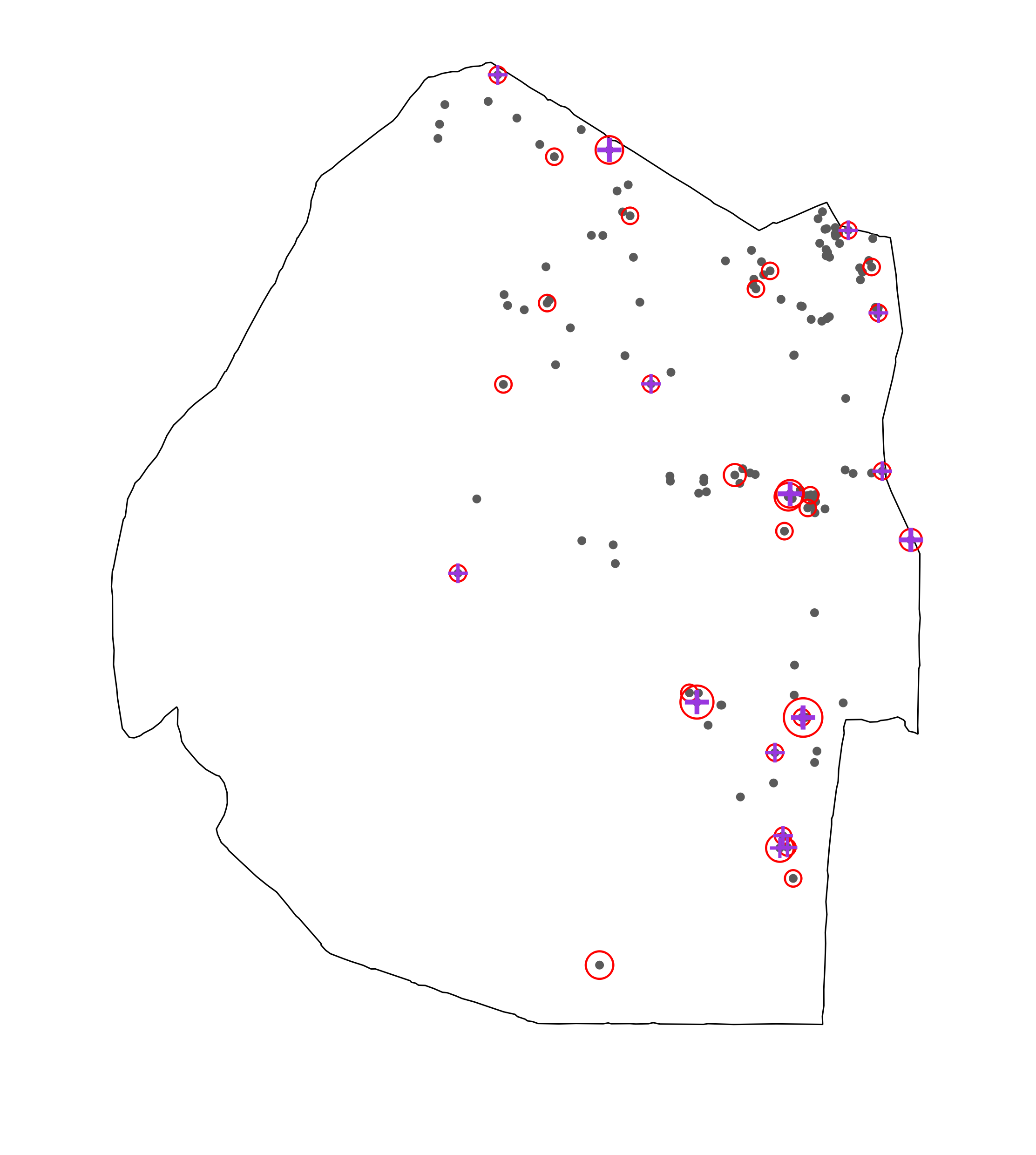

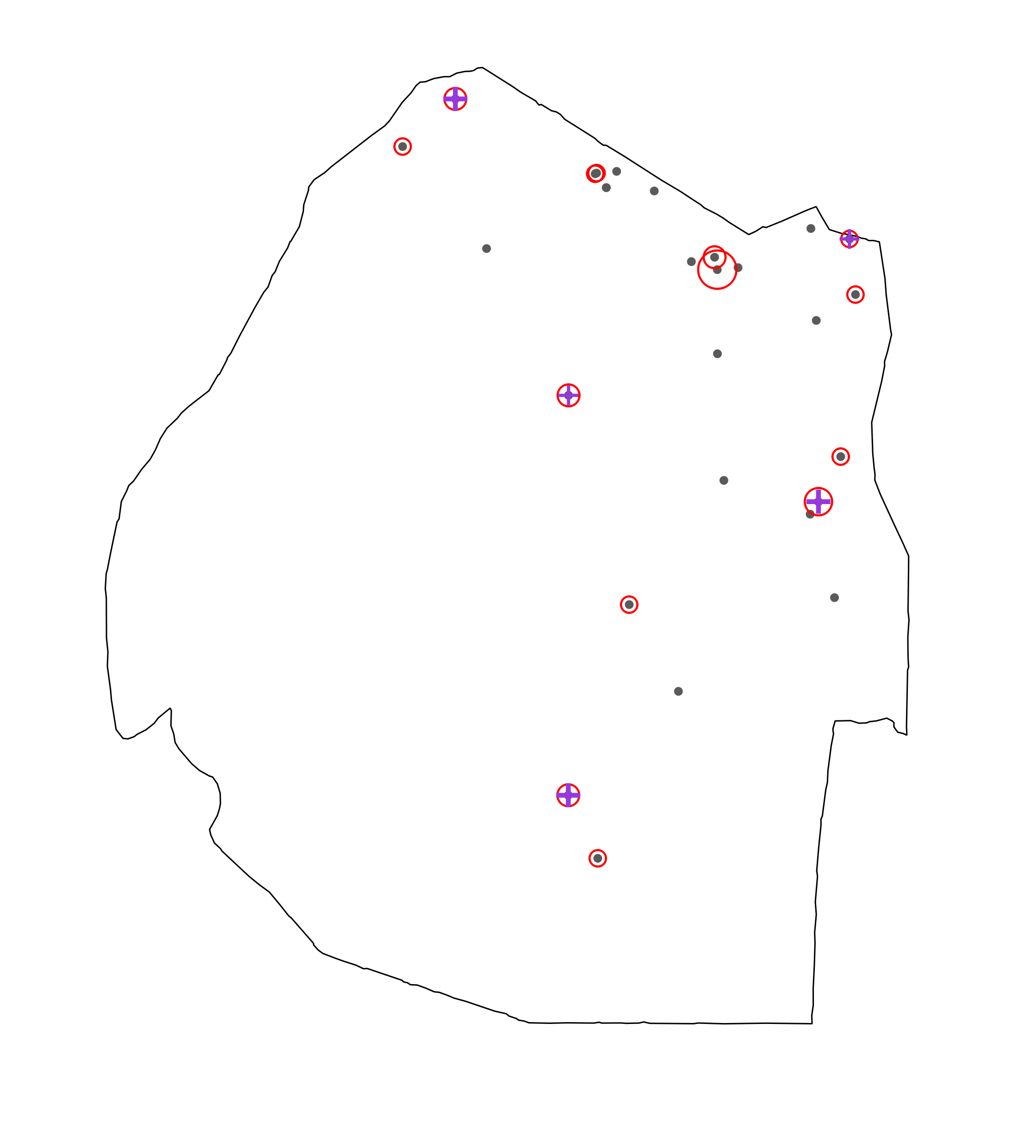

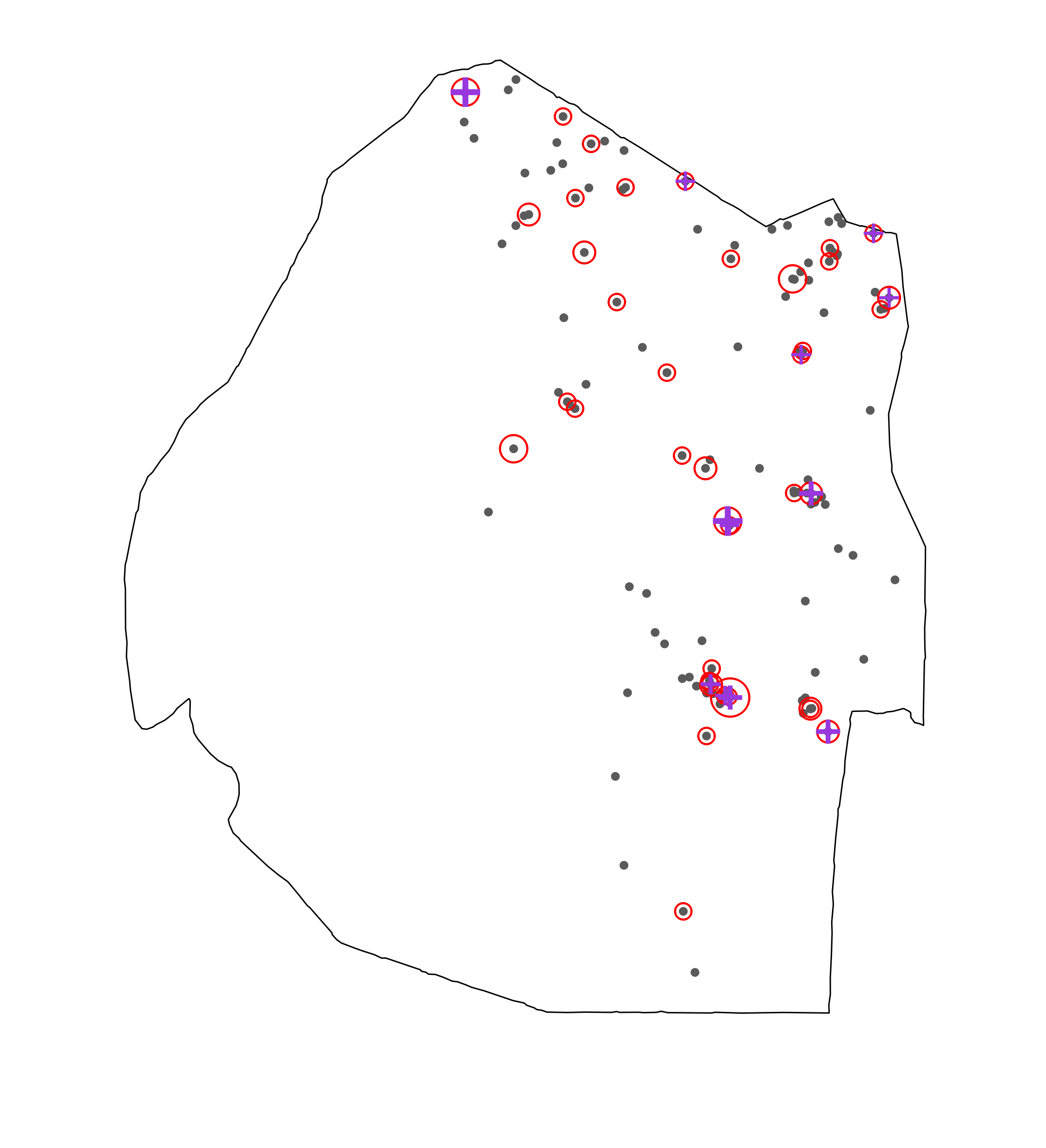


A. Year 1 High

B. Year 1 Low

C. Year 2 High

D. Year 2 Low

E. Year 3 High

F. Year 3 Low


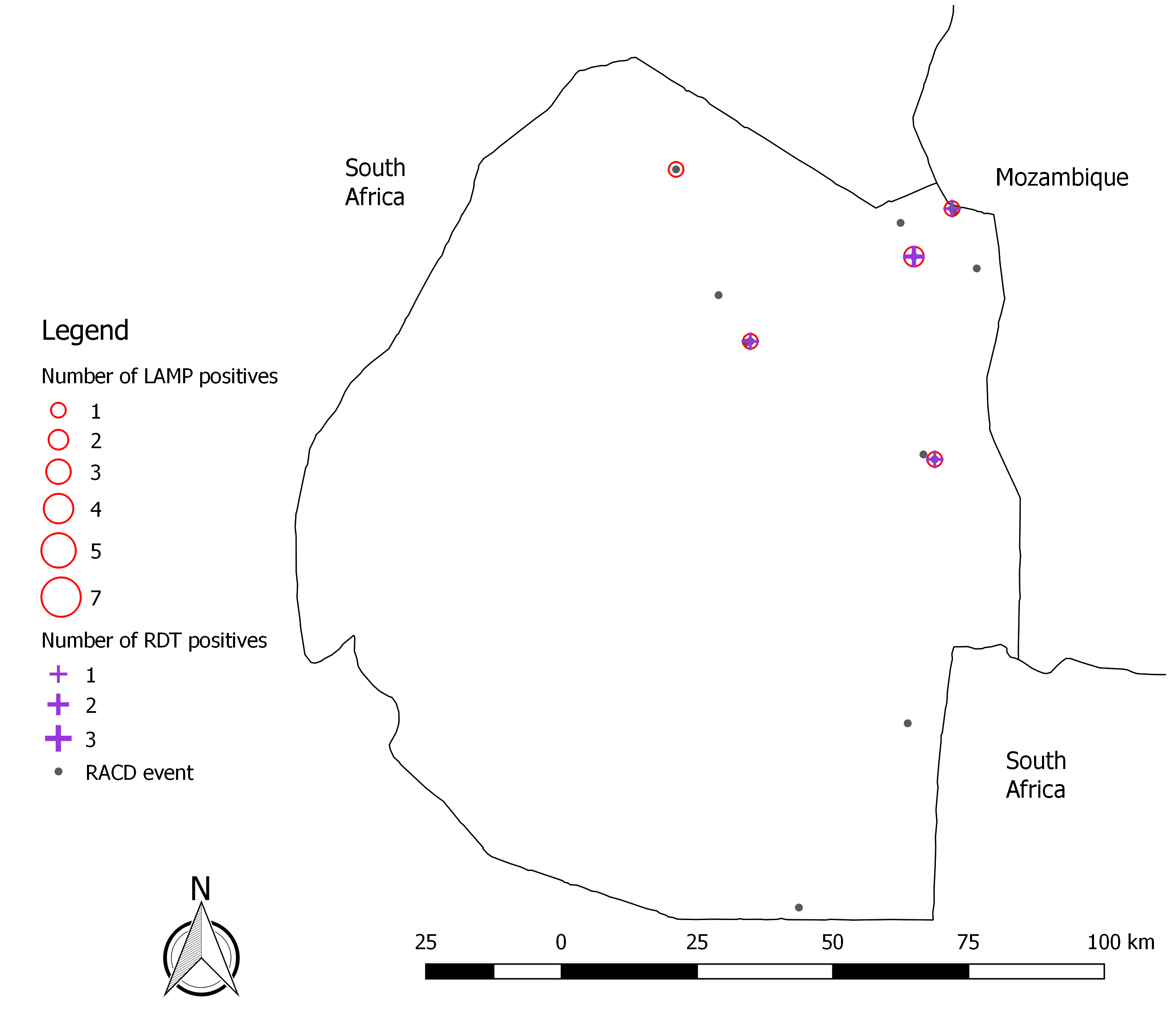


Supplemental figure 2. Maps of RACD events and RDT-positive and LAMP-positive infections detected by year and high versus low transmission season. RACD reactive case detection, RDT rapid diagnostic test, LAMP loop-mediated isothermal amplification
